# Dichlorido[(4*E*,11*E*)-5,7,12,14-tetra­benzyl-7,14-dimethyl-1,4,8,11-tetra­aza­cyclo­tetra­deca-4,11-diene]cobalt(III) perchlorate

**DOI:** 10.1107/S1600536811046484

**Published:** 2011-11-09

**Authors:** Tapashi G. Roy, Saroj K. S. Hazari, Kanak K. Barua, Seik Weng Ng, Edward R. T. Tiekink

**Affiliations:** a Department of Chemistry, University of Chittagong, Chittagong 4331, Bangladesh; b Department of Chemistry, University of Malaya, 50603 Kuala Lumpur, Malaysia; c Chemistry Department, Faculty of Science, King Abdulaziz University, PO Box 80203 Jeddah, Saudi Arabia

## Abstract

The Co^III^ atom in the title complex, [CoCl_2_(C_40_H_48_N_4_)]ClO_4_, is octa­hedrally coordinated within a *trans*-Cl_2_N_4_ donor set provided by the tetra­dentate macrocylic ligand and two chloride ions. The N—H atoms, which are orientated to one side of the N_4_ plane, form hydrogen bonds with chloride ions and perchlorate-O atoms. These along with C—H⋯O inter­actions consolidate the three-dimensional crystal structure. One of the benzene rings was disordered. This was resolved over two positions with the major component of the disorder having a site-occupancy factor of 0.672 (4).

## Related literature

For background to the synthesis, characterization, kinetic studies and biological activity of 14-membered methyl-substituted tetra­aza­macrocyclic ligands, their *N*-substituted derivatives and metal complexes, see: Bembi *et al.* (1990 ▶); Roy *et al.* (2007 ▶, 2011*a* ▶); Hazari *et al.* (2008 ▶). For a related structure, see: Roy *et al.* (2011*b* ▶).
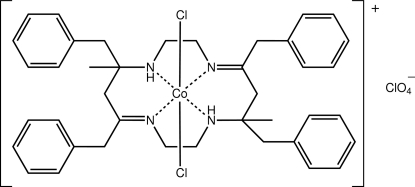


## Experimental

### 

#### Crystal data

[CoCl_2_(C_40_H_48_N_4_)]ClO_4_
*M* *_r_* = 814.10
Triclinic, 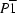

*a* = 10.8111 (7) Å
*b* = 13.835 (2) Å
*c* = 14.868 (3) Å
α = 73.66 (3)°
β = 70.06 (3)°
γ = 68.65 (2)°
*V* = 1915.6 (5) Å^3^
*Z* = 2
Mo *K*α radiation
μ = 0.70 mm^−1^
*T* = 153 K
0.30 × 0.20 × 0.10 mm

#### Data collection

Rigaku AFC12/SATURN724 diffractometer
Absorption correction: multi-scan (*ABSCOR*; Higashi, 1995 ▶) *T*_min_ = 0.627, *T*_max_ = 1.000
20336 measured reflections
7470 independent reflections
6940 reflections with *I* > 2σ(*I*)
*R*_int_ = 0.027

#### Refinement

*R*[*F* ^2^ > 2σ(*F* ^2^)] = 0.048
*wR*(*F* ^2^) = 0.142
*S* = 1.05
7470 reflections
476 parameters
2 restraints
H atoms treated by a mixture of independent and constrained refinement
Δρ_max_ = 0.72 e Å^−3^
Δρ_min_ = −0.50 e Å^−3^

### 

Data collection: *CrystalClear* (Rigaku/MSC, 2005 ▶); cell refinement: *CrystalClear*; data reduction: *CrystalClear*; program(s) used to solve structure: *PATTY* in *DIRDIF92* (Beurskens *et al.*, 1992 ▶); program(s) used to refine structure: *SHELXL97* (Sheldrick, 2008 ▶); molecular graphics: *ORTEP-3* (Farrugia, 1997 ▶) & *DIAMOND* (Brandenburg, 2006 ▶); software used to prepare material for publication: *publCIF* (Westrip, 2010 ▶).

## Supplementary Material

Crystal structure: contains datablock(s) global, I. DOI: 10.1107/S1600536811046484/pv2479sup1.cif

Structure factors: contains datablock(s) I. DOI: 10.1107/S1600536811046484/pv2479Isup2.hkl

Additional supplementary materials:  crystallographic information; 3D view; checkCIF report

## Figures and Tables

**Table 1:** Hydrogen-bond geometry (Å, °)

*D*—H⋯*A*	*D*—H	H⋯*A*	*D*⋯*A*	*D*—H⋯*A*
N2—H2n⋯O1	0.88 (3)	2.24 (3)	3.063 (4)	155 (2)
N4—H4n⋯Cl2^i^	0.88 (3)	2.64 (2)	3.432 (2)	150 (3)
C10—H10a⋯O3^ii^	0.99	2.50	3.437 (4)	159
C19—H19b⋯O1^iii^	0.99	2.54	3.409 (4)	147
C38a—H38a⋯O4^iv^	0.95	2.57	3.480 (3)	160

Symmetry codes: (i) 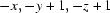; (ii) 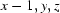; (iii) 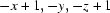; (iv) 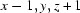.

**Fig. 1.**
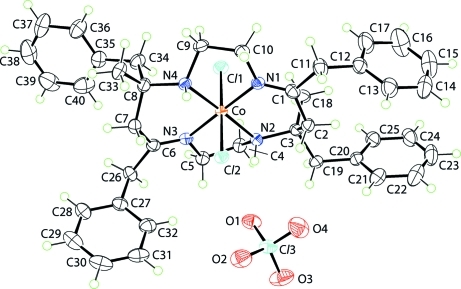
The molecular structure of (I) showing the atom-labelling scheme and displacement ellipsoids at the 50% probability level. Only the major orientation of the disordered C35—C40 ring is shown.

**Fig. 2.**
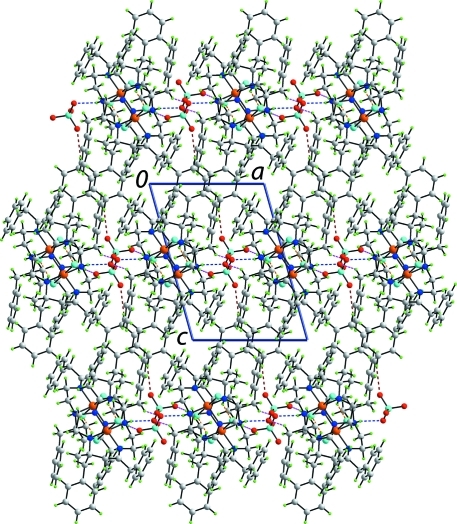
A view of the unit-cell contents in projection down the b axis in (I). The N—H···O(perchlorate), N—H···Cl, C—H···O(intra-layer) and C—H···O(inter-layer) interactions are shown as blue, orange, pink and brown dashed lines, respectively.

## supplementary crystallographic information

### Comment

In continuation of on-going studies of the synthesis, characterization and biological activities of substituted tetraazamacrocyclic ligands and their metal complexes (Bembi *et al.*, 1990; Roy *et al.*, 2007; Hazari *et al.*, 2008; Roy *et al.*, 2011*a*; Roy *et al.* 2011*b*), the synthesis and crystal structure of the title complex, (I), was investigated.

In (I), Fig. 1, the Co^III^ atom exists within a *trans*-Cl_2_N_4_ donor set defined by the four nitrogen atoms of the macrocyclic ligand and two chlorido atoms. The coordination geometry is based on an octahedron, with the greatest angular distortion manifested in the N2—Co—N3 angle of 83.81 (9)°. With respect to the central N_4_ plane, the rings adopt three distinct orientations. Two rings adopt similar orientations lying approximately perpendicular and parallel to the N_4_ plane: the dihedral angle between the N_4_ and the C12–C17 and C20–C25 planes are 86.928 (8) and 78.645 (10) °, respectively. The C27–C32 ring is also orientated in a perpendicular fashion (dihedral angle = 88.921 (10)°) but lies to one side of the N_4_ plane, with the C6—C26—C27—C28 torsion angle = 117.15 (4)°. The final ring is disordered over two positions. The major component is approximately planar with the N_4_ donor set, forming a dihedral angle of 20.644 (10)°, whereas the minor component forms a dihedral angle of 13.400 (9) °, *i.e*. even more co-planar. Within the N_4_ donor set, the two amine-H atoms are orientated to one side of the plane. The N2—H atom forms a contact with the perchlorate-O1 atom, and the N4—H forms an intramolecular N—H···Cl hydrogen bond, Table 2. These interactions along with several C—H···O contacts lead to the formation of supramolecular arrays in the *ab* plane. The layers stack along the *c* axis with the closest connection being of the type C—H···O, involving the perchlorate-O4 atom (Fig. 2 and Table 1).

### Experimental

The macrocyclic ligand, (4*E*,11*E*)-5,7,12,14-tetrabenzyl-7,14-dimethyl- 1,4,8,11-tetraazacyclotetradeca-4,11-diene (0.783 g, 1.0 mmol) was suspended in methanol (30 ml). Separately, cobaltous acetate (0.248 g, 1.0 mmol) was dissolved in methanol (30 ml). The combined solutions were heated on a water bath until the solution turned red. Concentrated HCl was added drop-wise so that the solution turned green. Then, about 1 ml HClO_4_ was added whereupon a green product started to appear. The mixture was heated in order to reduce the volume to 15 ml. The resulting solution was kept at room temperature for about 1 h. The solid product, (I), was separated by filtration, washed with dry ethanol, followed by diethylether and dried in a vacuum desiccator over silica-gel. The yield was about 50%. The same complex was also prepared by using the acetonitrile as the solvent instead of methanol. However, the yield was about 42%. Green crystals of (I) were isolated from the slow evaporation of its methanol solution.

### Refinement

The H-atoms were placed in calculated positions (C—H = 0.95–0.99 Å) and were included in the refinement in the riding model approximation, with *U*_iso_(H) = 1.2–1.5*U*_eq_(C). The N—H atoms were located from a difference map and refined with N—H = 0.88±0.01 Å, and with *U*_iso_(H)= 1.2*U*_equiv_(N). The C35–C40 phenyl ring was found to be disordered over two positions with a dihedral angle of 24.9 (3) Å between the orientations. After anisotropic refinement (pairs of atoms were constrained to have equivalent anisotropic displacement parameters), the major component had a site occupancy = 0.672 (4). A number of reflections, *i.e*. (3 2 11), (6 0 6), (10 5 0), (3 2 10), (2 1 12) and (2 2 11), were omitted from the final refinement owing to poor agreement.

### Crystal data

**Table:**

[CoCl_2_(C_40_H_48_N_4_)]ClO_4_	*Z* = 2
*M**_r_* = 814.10	*F*(000) = 852
Triclinic, *P*1	*D*_x_ = 1.411 Mg m^−^^3^
Hall symbol: -P 1	Mo *K*α radiation, λ = 0.71073 Å
*a* = 10.8111 (7) Å	Cell parameters from 6113 reflections
*b* = 13.835 (2) Å	θ = 2.2–30.4°
*c* = 14.868 (3) Å	µ = 0.70 mm^−^^1^
α = 73.66 (3)°	*T* = 153 K
β = 70.06 (3)°	Prism, green
γ = 68.65 (2)°	0.30 × 0.20 × 0.10 mm
*V* = 1915.6 (5) Å^3^	

### Data collection

**Table:**

Rigaku AFC12K/SATURN724 diffractometer	7470 independent reflections
Radiation source: fine-focus sealed tube	6940 reflections with *I* > 2σ(*I*)
graphite	*R*_int_ = 0.027
ω scans	θ_max_ = 26.0°, θ_min_ = 2.2°
Absorption correction: multi-scan (*ABSCOR*; Higashi, 1995)	*h* = −13→13
*T*_min_ = 0.627, *T*_max_ = 1.000	*k* = −15→17
20336 measured reflections	*l* = −18→18

### Refinement

**Table:**

Refinement on *F*^2^	Primary atom site location: structure-invariant direct methods
Least-squares matrix: full	Secondary atom site location: difference Fourier map
*R*[*F*^2^ > 2σ(*F*^2^)] = 0.048	Hydrogen site location: inferred from neighbouring sites
*wR*(*F*^2^) = 0.142	H atoms treated by a mixture of independent and constrained refinement
*S* = 1.05	*w* = 1/[σ^2^(*F*_o_^2^) + (0.0779*P*)^2^ + 2.2391*P*] where *P* = (*F*_o_^2^ + 2*F*_c_^2^)/3
7470 reflections	(Δ/σ)_max_ = 0.001
476 parameters	Δρ_max_ = 0.72 e Å^−^^3^
2 restraints	Δρ_min_ = −0.50 e Å^−^^3^

### Special details

**Table:**

Experimental. Microanalysis: Calculated for C_40_H_48_Cl_3_CoN_4_O_4_, C, 59.09; H, 5.78; N, 6.89; Co, 7.25%. Found, C, 59.25; H, 5.65; N, 6.89; Co, 7.05%. IR (cm^-1^): 3161 ν(N—H); 3024 ν(Ar—H); 2949 and 2978 ν(C—H); 1393 ν(CH_3_); 1095 and 622 ν(ClO_4_^-^); 550 ν(Co—N).
Geometry. All s.u.'s (except the s.u. in the dihedral angle between two l.s. planes) are estimated using the full covariance matrix. The cell s.u.'s are taken into account individually in the estimation of s.u.'s in distances, angles and torsion angles; correlations between s.u.'s in cell parameters are only used when they are defined by crystal symmetry. An approximate (isotropic) treatment of cell s.u.'s is used for estimating s.u.'s involving l.s. planes.
Refinement. Refinement of *F*^2^ against ALL reflections. The weighted *R*-factor *wR* and goodness of fit *S* are based on *F*^2^, conventional *R*-factors *R* are based on *F*, with *F* set to zero for negative *F*^2^. The threshold expression of *F*^2^ > 2σ(*F*^2^) is used only for calculating *R*-factors(gt) *etc*. and is not relevant to the choice of reflections for refinement. *R*-factors based on *F*^2^ are statistically about twice as large as those based on *F*, and *R*- factors based on ALL data will be even larger.

### Fractional atomic coordinates and isotropic or equivalent isotropic displacement parameters (Å^2^)

**Table:**

	*x*	*y*	*z*	*U*_iso_*/*U*_eq_	Occ. (<1)
Co	0.03217 (3)	0.25958 (2)	0.57912 (2)	0.01928 (12)	
Cl1	−0.08339 (7)	0.13989 (5)	0.64106 (5)	0.02986 (17)	
Cl2	0.14748 (6)	0.38188 (5)	0.51694 (4)	0.02299 (15)	
N1	−0.0314 (2)	0.29843 (17)	0.46384 (16)	0.0230 (4)	
N2	0.2055 (2)	0.15227 (16)	0.52957 (15)	0.0219 (4)	
H2N	0.265 (2)	0.186 (2)	0.518 (2)	0.026*	
N3	0.0985 (2)	0.22239 (16)	0.69411 (16)	0.0227 (4)	
N4	−0.1336 (2)	0.37339 (17)	0.62547 (15)	0.0217 (4)	
H4N	−0.105 (3)	0.4285 (16)	0.5924 (19)	0.026*	
C1	0.0414 (3)	0.2780 (2)	0.3799 (2)	0.0272 (6)	
C2	0.1919 (3)	0.2166 (2)	0.36292 (19)	0.0263 (5)	
H2A	0.2430	0.2636	0.3621	0.032*	
H2B	0.2250	0.1975	0.2976	0.032*	
C3	0.2289 (3)	0.1159 (2)	0.43667 (19)	0.0235 (5)	
C4	0.2349 (3)	0.0693 (2)	0.61436 (19)	0.0268 (6)	
H4A	0.3298	0.0215	0.5966	0.032*	
H4B	0.1696	0.0271	0.6367	0.032*	
C5	0.2186 (3)	0.1249 (2)	0.69361 (19)	0.0269 (6)	
H5A	0.2038	0.0775	0.7575	0.032*	
H5B	0.3034	0.1438	0.6821	0.032*	
C6	0.0646 (3)	0.2809 (2)	0.75664 (19)	0.0248 (5)	
C7	−0.0518 (3)	0.3823 (2)	0.75592 (19)	0.0258 (5)	
H7A	−0.0141	0.4381	0.7091	0.031*	
H7B	−0.0823	0.4014	0.8211	0.031*	
C8	−0.1801 (3)	0.3860 (2)	0.73077 (19)	0.0264 (6)	
C9	−0.2421 (3)	0.3776 (2)	0.5824 (2)	0.0279 (6)	
H9A	−0.3176	0.4446	0.5888	0.033*	
H9B	−0.2811	0.3183	0.6163	0.033*	
C10	−0.1742 (3)	0.3700 (2)	0.4760 (2)	0.0261 (5)	
H10A	−0.2277	0.3430	0.4518	0.031*	
H10B	−0.1729	0.4410	0.4375	0.031*	
C11	−0.0180 (3)	0.3201 (3)	0.2918 (2)	0.0397 (7)	
H11A	−0.1191	0.3332	0.3150	0.048*	
H11B	−0.0006	0.3886	0.2589	0.048*	
C12	0.0400 (3)	0.2479 (2)	0.2186 (2)	0.0331 (6)	
C13	0.1397 (4)	0.2649 (3)	0.1340 (2)	0.0432 (8)	
H13	0.1690	0.3257	0.1200	0.052*	
C14	0.1982 (4)	0.1960 (3)	0.0691 (2)	0.0521 (9)	
H14	0.2669	0.2094	0.0112	0.063*	
C15	0.1565 (5)	0.1085 (3)	0.0888 (3)	0.0630 (12)	
H15	0.1967	0.0604	0.0447	0.076*	
C16	0.0565 (6)	0.0897 (3)	0.1725 (3)	0.0714 (13)	
H16	0.0278	0.0287	0.1860	0.086*	
C17	−0.0029 (4)	0.1600 (3)	0.2374 (2)	0.0534 (9)	
H17	−0.0729	0.1473	0.2947	0.064*	
C18	0.1422 (3)	0.0454 (2)	0.4501 (2)	0.0305 (6)	
H18A	0.1701	−0.0196	0.4958	0.046*	
H18B	0.1556	0.0280	0.3873	0.046*	
H18C	0.0447	0.0824	0.4758	0.046*	
C19	0.3844 (3)	0.0560 (2)	0.4032 (2)	0.0274 (6)	
H19A	0.4384	0.1015	0.4010	0.033*	
H19B	0.4063	−0.0079	0.4528	0.033*	
C20	0.4313 (3)	0.0230 (2)	0.3052 (2)	0.0281 (6)	
C21	0.4946 (3)	0.0826 (3)	0.2235 (2)	0.0398 (7)	
H21	0.5067	0.1452	0.2289	0.048*	
C22	0.5411 (4)	0.0521 (3)	0.1329 (3)	0.0528 (9)	
H22	0.5844	0.0938	0.0771	0.063*	
C23	0.5241 (4)	−0.0387 (3)	0.1248 (3)	0.0519 (10)	
H23	0.5557	−0.0598	0.0633	0.062*	
C24	0.4615 (3)	−0.0988 (3)	0.2055 (3)	0.0451 (8)	
H24	0.4493	−0.1611	0.1995	0.054*	
C25	0.4161 (3)	−0.0692 (2)	0.2956 (2)	0.0349 (6)	
H25	0.3743	−0.1119	0.3512	0.042*	
C26	0.1434 (3)	0.2615 (2)	0.8307 (2)	0.0298 (6)	
H26A	0.0801	0.2578	0.8971	0.036*	
H26B	0.2173	0.1933	0.8275	0.036*	
C27	0.2058 (3)	0.3502 (2)	0.8096 (2)	0.0291 (6)	
C28	0.1666 (3)	0.4162 (2)	0.8764 (2)	0.0355 (7)	
H28	0.0996	0.4058	0.9364	0.043*	
C29	0.2248 (4)	0.4971 (3)	0.8560 (3)	0.0450 (8)	
H29	0.1979	0.5414	0.9022	0.054*	
C30	0.3218 (3)	0.5132 (3)	0.7689 (3)	0.0441 (8)	
H30	0.3616	0.5683	0.7552	0.053*	
C31	0.3605 (3)	0.4491 (3)	0.7019 (3)	0.0388 (7)	
H31	0.4265	0.4607	0.6417	0.047*	
C32	0.3035 (3)	0.3676 (2)	0.7217 (2)	0.0340 (6)	
H32	0.3312	0.3235	0.6752	0.041*	
C33	−0.2828 (3)	0.4958 (2)	0.7397 (2)	0.0336 (6)	
H33A	−0.3678	0.4998	0.7268	0.050*	
H33B	−0.2427	0.5491	0.6924	0.050*	
H33C	−0.3038	0.5088	0.8055	0.050*	
Cl3	0.54134 (7)	0.26751 (5)	0.43796 (5)	0.03305 (18)	
O1	0.4658 (2)	0.20304 (16)	0.51435 (16)	0.0382 (5)	
O2	0.5109 (3)	0.36627 (18)	0.4651 (2)	0.0499 (6)	
O3	0.6847 (2)	0.2140 (2)	0.4224 (2)	0.0592 (7)	
O4	0.4999 (3)	0.2853 (2)	0.35077 (18)	0.0575 (7)	
C34A	−0.2460 (18)	0.2928 (8)	0.8000 (11)	0.0270 (17)	0.672 (3)
H34A	−0.3205	0.2923	0.7758	0.032*	0.672 (3)
H34B	−0.1740	0.2238	0.7954	0.032*	0.672 (3)
C35A	−0.3028 (3)	0.3053 (3)	0.90364 (18)	0.0331 (9)	0.672 (3)
C36A	−0.4428 (3)	0.3556 (3)	0.9363 (2)	0.0439 (11)	0.672 (3)
H36A	−0.4986	0.3835	0.8922	0.053*	0.672 (3)
C37A	−0.5011 (3)	0.3649 (3)	1.0337 (2)	0.0550 (13)	0.672 (3)
H37A	−0.5968	0.3993	1.0560	0.066*	0.672 (3)
C38A	−0.4194 (4)	0.3240 (3)	1.09829 (16)	0.0597 (16)	0.672 (3)
H38A	−0.4593	0.3304	1.1648	0.072*	0.672 (3)
C39A	−0.2795 (4)	0.2738 (3)	1.0656 (2)	0.0600 (16)	0.672 (3)
H39A	−0.2236	0.2458	1.1098	0.072*	0.672 (3)
C40A	−0.2212 (3)	0.2644 (3)	0.9683 (2)	0.0477 (13)	0.672 (3)
H40A	−0.1255	0.2300	0.9459	0.057*	0.672 (3)
C34B	−0.245 (4)	0.314 (2)	0.789 (3)	0.0270 (17)	0.328 (3)
H34C	−0.3364	0.3354	0.7759	0.032*	0.328 (3)
H34D	−0.1923	0.2458	0.7662	0.032*	0.328 (3)
C35B	−0.2705 (8)	0.2912 (6)	0.9041 (4)	0.0331 (9)	0.328 (3)
C36B	−0.3766 (7)	0.3593 (5)	0.9616 (5)	0.0439 (11)	0.328 (3)
H36B	−0.4417	0.4163	0.9335	0.053*	0.328 (3)
C37B	−0.3876 (8)	0.3441 (6)	1.0601 (5)	0.0550 (13)	0.328 (3)
H37B	−0.4601	0.3907	1.0994	0.066*	0.328 (3)
C38B	−0.2923 (10)	0.2607 (7)	1.1012 (4)	0.0597 (16)	0.328 (3)
H38B	−0.2998	0.2503	1.1686	0.07 (4)*	0.328 (3)
C39B	−0.1861 (8)	0.1926 (6)	1.0438 (5)	0.0600 (16)	0.328 (3)
H39B	−0.1210	0.1356	1.0719	0.072*	0.328 (3)
C40B	−0.1752 (7)	0.2078 (6)	0.9452 (5)	0.0477 (13)	0.328 (3)
H40B	−0.1027	0.1613	0.9060	0.057*	0.328 (3)

### Atomic displacement parameters (Å^2^)

**Table:**

	*U*^11^	*U*^22^	*U*^33^	*U*^12^	*U*^13^	*U*^23^
Co	0.01748 (19)	0.0189 (2)	0.02131 (19)	−0.00528 (14)	−0.00556 (14)	−0.00311 (13)
Cl1	0.0289 (3)	0.0277 (3)	0.0344 (4)	−0.0131 (3)	−0.0044 (3)	−0.0063 (3)
Cl2	0.0211 (3)	0.0206 (3)	0.0262 (3)	−0.0075 (2)	−0.0044 (2)	−0.0033 (2)
N1	0.0188 (10)	0.0235 (11)	0.0259 (11)	−0.0040 (8)	−0.0070 (9)	−0.0053 (9)
N2	0.0227 (11)	0.0197 (10)	0.0241 (11)	−0.0051 (8)	−0.0084 (9)	−0.0041 (8)
N3	0.0197 (10)	0.0214 (11)	0.0266 (11)	−0.0068 (8)	−0.0066 (9)	−0.0022 (8)
N4	0.0195 (10)	0.0238 (11)	0.0209 (10)	−0.0072 (8)	−0.0039 (8)	−0.0034 (8)
C1	0.0277 (14)	0.0248 (13)	0.0302 (14)	−0.0046 (10)	−0.0121 (11)	−0.0055 (11)
C2	0.0238 (13)	0.0283 (14)	0.0251 (13)	−0.0045 (10)	−0.0056 (11)	−0.0075 (11)
C3	0.0206 (12)	0.0228 (13)	0.0286 (13)	−0.0025 (10)	−0.0090 (11)	−0.0093 (10)
C4	0.0270 (13)	0.0225 (13)	0.0273 (13)	−0.0023 (10)	−0.0099 (11)	−0.0023 (10)
C5	0.0255 (13)	0.0254 (13)	0.0267 (13)	−0.0023 (10)	−0.0106 (11)	−0.0026 (10)
C6	0.0247 (13)	0.0274 (13)	0.0236 (12)	−0.0118 (10)	−0.0064 (10)	−0.0012 (10)
C7	0.0289 (14)	0.0254 (13)	0.0229 (12)	−0.0085 (11)	−0.0045 (11)	−0.0063 (10)
C8	0.0247 (13)	0.0288 (14)	0.0233 (13)	−0.0063 (11)	−0.0034 (11)	−0.0071 (10)
C9	0.0175 (12)	0.0323 (14)	0.0323 (14)	−0.0053 (10)	−0.0062 (11)	−0.0064 (11)
C10	0.0202 (13)	0.0262 (13)	0.0317 (14)	−0.0016 (10)	−0.0110 (11)	−0.0071 (11)
C11	0.0395 (17)	0.0425 (17)	0.0331 (16)	0.0050 (13)	−0.0184 (14)	−0.0131 (13)
C12	0.0319 (15)	0.0384 (16)	0.0264 (14)	−0.0027 (12)	−0.0128 (12)	−0.0058 (12)
C13	0.0477 (19)	0.0477 (19)	0.0307 (16)	−0.0109 (15)	−0.0127 (14)	−0.0036 (14)
C14	0.054 (2)	0.058 (2)	0.0284 (16)	−0.0016 (17)	−0.0061 (15)	−0.0110 (15)
C15	0.093 (3)	0.047 (2)	0.039 (2)	0.000 (2)	−0.023 (2)	−0.0148 (17)
C16	0.125 (4)	0.056 (3)	0.048 (2)	−0.045 (3)	−0.026 (3)	−0.0022 (19)
C17	0.070 (3)	0.070 (3)	0.0291 (16)	−0.036 (2)	−0.0096 (17)	−0.0064 (16)
C18	0.0267 (14)	0.0287 (14)	0.0398 (15)	−0.0083 (11)	−0.0094 (12)	−0.0109 (12)
C19	0.0214 (13)	0.0280 (14)	0.0320 (14)	−0.0033 (10)	−0.0078 (11)	−0.0087 (11)
C20	0.0196 (12)	0.0307 (14)	0.0306 (14)	0.0020 (10)	−0.0076 (11)	−0.0118 (11)
C21	0.0331 (16)	0.0392 (17)	0.0391 (17)	−0.0060 (13)	−0.0028 (13)	−0.0097 (13)
C22	0.045 (2)	0.057 (2)	0.0339 (17)	−0.0023 (16)	0.0015 (15)	−0.0065 (16)
C23	0.046 (2)	0.064 (2)	0.0354 (17)	0.0091 (17)	−0.0118 (15)	−0.0256 (17)
C24	0.0412 (18)	0.0457 (19)	0.050 (2)	0.0034 (15)	−0.0164 (16)	−0.0280 (16)
C25	0.0323 (15)	0.0325 (15)	0.0393 (16)	−0.0025 (12)	−0.0100 (13)	−0.0144 (13)
C26	0.0348 (15)	0.0318 (14)	0.0255 (13)	−0.0103 (12)	−0.0115 (12)	−0.0044 (11)
C27	0.0285 (14)	0.0307 (14)	0.0303 (14)	−0.0081 (11)	−0.0144 (12)	−0.0018 (11)
C28	0.0411 (17)	0.0379 (16)	0.0324 (15)	−0.0121 (13)	−0.0155 (13)	−0.0061 (12)
C29	0.057 (2)	0.0374 (17)	0.054 (2)	−0.0145 (15)	−0.0316 (18)	−0.0073 (15)
C30	0.0443 (18)	0.0365 (17)	0.063 (2)	−0.0184 (14)	−0.0333 (17)	0.0064 (15)
C31	0.0281 (15)	0.0401 (17)	0.0474 (18)	−0.0133 (13)	−0.0167 (14)	0.0059 (14)
C32	0.0292 (15)	0.0380 (16)	0.0352 (15)	−0.0082 (12)	−0.0139 (12)	−0.0027 (12)
C33	0.0285 (14)	0.0323 (15)	0.0311 (14)	−0.0018 (11)	−0.0012 (12)	−0.0103 (12)
Cl3	0.0299 (4)	0.0291 (4)	0.0425 (4)	−0.0119 (3)	−0.0124 (3)	−0.0022 (3)
O1	0.0405 (12)	0.0344 (11)	0.0437 (12)	−0.0187 (9)	−0.0179 (10)	0.0050 (9)
O2	0.0455 (13)	0.0350 (12)	0.0713 (17)	−0.0174 (10)	−0.0075 (12)	−0.0154 (11)
O3	0.0303 (12)	0.0483 (15)	0.093 (2)	−0.0083 (11)	−0.0085 (13)	−0.0182 (14)
O4	0.0720 (18)	0.0681 (17)	0.0413 (14)	−0.0345 (14)	−0.0223 (13)	0.0043 (12)
C34A	0.0324 (16)	0.021 (5)	0.023 (4)	−0.018 (4)	0.001 (2)	0.005 (4)
C35A	0.038 (3)	0.0297 (19)	0.0295 (15)	−0.0150 (18)	−0.0039 (16)	−0.0024 (13)
C36A	0.041 (3)	0.046 (2)	0.033 (2)	−0.011 (2)	0.0013 (19)	−0.0047 (19)
C37A	0.061 (3)	0.051 (3)	0.035 (2)	−0.015 (3)	0.011 (2)	−0.012 (2)
C38A	0.097 (5)	0.060 (4)	0.029 (2)	−0.051 (3)	0.002 (3)	−0.005 (2)
C39A	0.079 (4)	0.082 (4)	0.037 (3)	−0.054 (3)	−0.022 (3)	0.011 (3)
C40A	0.042 (3)	0.053 (3)	0.043 (3)	−0.023 (3)	−0.010 (2)	0.010 (2)
C34B	0.0324 (16)	0.021 (5)	0.023 (4)	−0.018 (4)	0.001 (2)	0.005 (4)
C35B	0.038 (3)	0.0297 (19)	0.0295 (15)	−0.0150 (18)	−0.0039 (16)	−0.0024 (13)
C36B	0.041 (3)	0.046 (2)	0.033 (2)	−0.011 (2)	0.0013 (19)	−0.0047 (19)
C37B	0.061 (3)	0.051 (3)	0.035 (2)	−0.015 (3)	0.011 (2)	−0.012 (2)
C38B	0.097 (5)	0.060 (4)	0.029 (2)	−0.051 (3)	0.002 (3)	−0.005 (2)
C39B	0.079 (4)	0.082 (4)	0.037 (3)	−0.054 (3)	−0.022 (3)	0.011 (3)
C40B	0.042 (3)	0.053 (3)	0.043 (3)	−0.023 (3)	−0.010 (2)	0.010 (2)

### Geometric parameters (Å, °)

**Table:**

Co—N1	1.927 (2)	C20—C21	1.381 (4)
Co—N3	1.943 (2)	C20—C25	1.394 (4)
Co—N4	1.969 (2)	C21—C22	1.396 (5)
Co—N2	1.977 (2)	C21—H21	0.9500
Co—Cl1	2.2395 (9)	C22—C23	1.377 (6)
Co—Cl2	2.2676 (8)	C22—H22	0.9500
N1—C1	1.274 (4)	C23—C24	1.373 (5)
N1—C10	1.482 (3)	C23—H23	0.9500
N2—C4	1.484 (3)	C24—C25	1.384 (4)
N2—C3	1.511 (3)	C24—H24	0.9500
N2—H2N	0.877 (10)	C25—H25	0.9500
N3—C6	1.276 (3)	C26—C27	1.519 (4)
N3—C5	1.493 (3)	C26—H26A	0.9900
N4—C9	1.494 (3)	C26—H26B	0.9900
N4—C8	1.512 (3)	C27—C28	1.393 (4)
N4—H4N	0.878 (10)	C27—C32	1.396 (4)
C1—C2	1.506 (4)	C28—C29	1.390 (5)
C1—C11	1.530 (4)	C28—H28	0.9500
C2—C3	1.529 (4)	C29—C30	1.381 (5)
C2—H2A	0.9900	C29—H29	0.9500
C2—H2B	0.9900	C30—C31	1.377 (5)
C3—C18	1.517 (4)	C30—H30	0.9500
C3—C19	1.552 (3)	C31—C32	1.390 (4)
C4—C5	1.511 (4)	C31—H31	0.9500
C4—H4A	0.9900	C32—H32	0.9500
C4—H4B	0.9900	C33—H33A	0.9800
C5—H5A	0.9900	C33—H33B	0.9800
C5—H5B	0.9900	C33—H33C	0.9800
C6—C7	1.506 (4)	Cl3—O3	1.421 (2)
C6—C26	1.522 (4)	Cl3—O2	1.425 (2)
C7—C8	1.535 (4)	Cl3—O4	1.441 (3)
C7—H7A	0.9900	Cl3—O1	1.442 (2)
C7—H7B	0.9900	C34A—C35A	1.486 (17)
C8—C33	1.531 (4)	C34A—H34A	0.9900
C8—C34B	1.36 (3)	C34A—H34B	0.9900
C8—C34A	1.629 (10)	C35A—C36A	1.3900
C9—C10	1.515 (4)	C35A—C40A	1.3900
C9—H9A	0.9900	C36A—C37A	1.3900
C9—H9B	0.9900	C36A—H36A	0.9500
C10—H10A	0.9900	C37A—C38A	1.3900
C10—H10B	0.9900	C37A—H37A	0.9500
C11—C12	1.505 (4)	C38A—C39A	1.3900
C11—H11A	0.9900	C38A—H38A	0.9500
C11—H11B	0.9900	C39A—C40A	1.3900
C12—C13	1.375 (4)	C39A—H39A	0.9500
C12—C17	1.379 (5)	C40A—H40A	0.9500
C13—C14	1.380 (5)	C34B—C35B	1.60 (4)
C13—H13	0.9500	C34B—H34C	0.9900
C14—C15	1.365 (6)	C34B—H34D	0.9900
C14—H14	0.9500	C35B—C36B	1.3900
C15—C16	1.374 (6)	C35B—C40B	1.3900
C15—H15	0.9500	C36B—C37B	1.3900
C16—C17	1.393 (6)	C36B—H36B	0.9500
C16—H16	0.9500	C37B—C38B	1.3900
C17—H17	0.9500	C37B—H37B	0.9500
C18—H18A	0.9800	C38B—C39B	1.3900
C18—H18B	0.9800	C38B—H38B	0.9500
C18—H18C	0.9800	C39B—C40B	1.3900
C19—C20	1.514 (4)	C39B—H39B	0.9500
C19—H19A	0.9900	C40B—H40B	0.9500
C19—H19B	0.9900		
			
N1—Co—N3	178.83 (9)	C3—C18—H18A	109.5
N1—Co—N4	84.22 (9)	C3—C18—H18B	109.5
N3—Co—N4	95.83 (9)	H18A—C18—H18B	109.5
N1—Co—N2	96.07 (9)	C3—C18—H18C	109.5
N3—Co—N2	83.80 (9)	H18A—C18—H18C	109.5
N4—Co—N2	176.17 (9)	H18B—C18—H18C	109.5
N1—Co—Cl1	89.85 (7)	C20—C19—C3	115.3 (2)
N3—Co—Cl1	91.32 (7)	C20—C19—H19A	108.4
N4—Co—Cl1	91.81 (7)	C3—C19—H19A	108.4
N2—Co—Cl1	92.00 (7)	C20—C19—H19B	108.4
N1—Co—Cl2	90.11 (7)	C3—C19—H19B	108.4
N3—Co—Cl2	88.72 (7)	H19A—C19—H19B	107.5
N4—Co—Cl2	87.68 (7)	C21—C20—C25	118.6 (3)
N2—Co—Cl2	88.50 (7)	C21—C20—C19	120.0 (3)
Cl1—Co—Cl2	179.50 (3)	C25—C20—C19	121.3 (3)
C1—N1—C10	119.7 (2)	C20—C21—C22	120.8 (3)
C1—N1—Co	126.26 (18)	C20—C21—H21	119.6
C10—N1—Co	113.59 (17)	C22—C21—H21	119.6
C4—N2—C3	117.1 (2)	C23—C22—C21	119.7 (3)
C4—N2—Co	106.90 (16)	C23—C22—H22	120.2
C3—N2—Co	119.99 (15)	C21—C22—H22	120.2
C4—N2—H2N	103 (2)	C24—C23—C22	120.0 (3)
C3—N2—H2N	107 (2)	C24—C23—H23	120.0
Co—N2—H2N	100 (2)	C22—C23—H23	120.0
C6—N3—C5	120.0 (2)	C23—C24—C25	120.4 (3)
C6—N3—Co	126.05 (18)	C23—C24—H24	119.8
C5—N3—Co	112.83 (16)	C25—C24—H24	119.8
C9—N4—C8	116.6 (2)	C24—C25—C20	120.4 (3)
C9—N4—Co	107.52 (16)	C24—C25—H25	119.8
C8—N4—Co	120.35 (16)	C20—C25—H25	119.8
C9—N4—H4N	105 (2)	C27—C26—C6	109.7 (2)
C8—N4—H4N	105 (2)	C27—C26—H26A	109.7
Co—N4—H4N	100 (2)	C6—C26—H26A	109.7
N1—C1—C2	120.8 (2)	C27—C26—H26B	109.7
N1—C1—C11	121.6 (2)	C6—C26—H26B	109.7
C2—C1—C11	117.5 (2)	H26A—C26—H26B	108.2
C1—C2—C3	116.3 (2)	C28—C27—C32	118.8 (3)
C1—C2—H2A	108.2	C28—C27—C26	121.1 (3)
C3—C2—H2A	108.2	C32—C27—C26	120.1 (3)
C1—C2—H2B	108.2	C29—C28—C27	120.4 (3)
C3—C2—H2B	108.2	C29—C28—H28	119.8
H2A—C2—H2B	107.4	C27—C28—H28	119.8
N2—C3—C18	112.3 (2)	C30—C29—C28	120.2 (3)
N2—C3—C2	105.6 (2)	C30—C29—H29	119.9
C18—C3—C2	111.0 (2)	C28—C29—H29	119.9
N2—C3—C19	107.4 (2)	C31—C30—C29	119.9 (3)
C18—C3—C19	110.3 (2)	C31—C30—H30	120.0
C2—C3—C19	109.9 (2)	C29—C30—H30	120.0
N2—C4—C5	106.9 (2)	C30—C31—C32	120.4 (3)
N2—C4—H4A	110.3	C30—C31—H31	119.8
C5—C4—H4A	110.3	C32—C31—H31	119.8
N2—C4—H4B	110.3	C31—C32—C27	120.3 (3)
C5—C4—H4B	110.3	C31—C32—H32	119.9
H4A—C4—H4B	108.6	C27—C32—H32	119.9
N3—C5—C4	109.4 (2)	C8—C33—H33A	109.5
N3—C5—H5A	109.8	C8—C33—H33B	109.5
C4—C5—H5A	109.8	H33A—C33—H33B	109.5
N3—C5—H5B	109.8	C8—C33—H33C	109.5
C4—C5—H5B	109.8	H33A—C33—H33C	109.5
H5A—C5—H5B	108.2	H33B—C33—H33C	109.5
N3—C6—C7	121.2 (2)	O3—Cl3—O2	109.85 (16)
N3—C6—C26	124.3 (2)	O3—Cl3—O4	110.38 (19)
C7—C6—C26	114.3 (2)	O2—Cl3—O4	109.29 (17)
C6—C7—C8	118.6 (2)	O3—Cl3—O1	108.94 (15)
C6—C7—H7A	107.7	O2—Cl3—O1	109.77 (15)
C8—C7—H7A	107.7	O4—Cl3—O1	108.59 (14)
C6—C7—H7B	107.7	C35A—C34A—C8	113.0 (10)
C8—C7—H7B	107.7	C35A—C34A—H34A	109.0
H7A—C7—H7B	107.1	C8—C34A—H34A	109.0
N4—C8—C33	108.7 (2)	C35A—C34A—H34B	109.0
N4—C8—C7	106.2 (2)	C8—C34A—H34B	109.0
C33—C8—C7	107.4 (2)	H34A—C34A—H34B	107.8
N4—C8—C34B	111.2 (18)	C36A—C35A—C40A	120.0
C33—C8—C34B	107.7 (16)	C36A—C35A—C34A	118.2 (7)
C7—C8—C34B	115.3 (17)	C40A—C35A—C34A	121.8 (7)
N4—C8—C34A	110.5 (7)	C35A—C36A—C37A	120.0
C33—C8—C34A	112.0 (6)	C35A—C36A—H36A	120.0
C7—C8—C34A	111.8 (6)	C37A—C36A—H36A	120.0
N4—C9—C10	107.3 (2)	C38A—C37A—C36A	120.0
N4—C9—H9A	110.3	C38A—C37A—H37A	120.0
C10—C9—H9A	110.3	C36A—C37A—H37A	120.0
N4—C9—H9B	110.3	C37A—C38A—C39A	120.0
C10—C9—H9B	110.3	C37A—C38A—H38A	120.0
H9A—C9—H9B	108.5	C39A—C38A—H38A	120.0
N1—C10—C9	110.0 (2)	C38A—C39A—C40A	120.0
N1—C10—H10A	109.7	C38A—C39A—H39A	120.0
C9—C10—H10A	109.7	C40A—C39A—H39A	120.0
N1—C10—H10B	109.7	C39A—C40A—C35A	120.0
C9—C10—H10B	109.7	C39A—C40A—H40A	120.0
H10A—C10—H10B	108.2	C35A—C40A—H40A	120.0
C12—C11—C1	114.6 (2)	C35B—C34B—C8	121 (3)
C12—C11—H11A	108.6	C35B—C34B—H34C	107.1
C1—C11—H11A	108.6	C8—C34B—H34C	107.1
C12—C11—H11B	108.6	C35B—C34B—H34D	107.1
C1—C11—H11B	108.6	C8—C34B—H34D	107.1
H11A—C11—H11B	107.6	H34C—C34B—H34D	106.8
C13—C12—C17	118.5 (3)	C36B—C35B—C40B	120.0
C13—C12—C11	121.5 (3)	C36B—C35B—C34B	121.1 (14)
C17—C12—C11	119.9 (3)	C40B—C35B—C34B	118.5 (14)
C12—C13—C14	121.7 (4)	C37B—C36B—C35B	120.0
C12—C13—H13	119.1	C37B—C36B—H36B	120.0
C14—C13—H13	119.1	C35B—C36B—H36B	120.0
C15—C14—C13	119.4 (4)	C36B—C37B—C38B	120.0
C15—C14—H14	120.3	C36B—C37B—H37B	120.0
C13—C14—H14	120.3	C38B—C37B—H37B	120.0
C14—C15—C16	120.1 (4)	C39B—C38B—C37B	120.0
C14—C15—H15	119.9	C39B—C38B—H38B	120.0
C16—C15—H15	119.9	C37B—C38B—H38B	120.0
C15—C16—C17	120.2 (4)	C38B—C39B—C40B	120.0
C15—C16—H16	119.9	C38B—C39B—H39B	120.0
C17—C16—H16	119.9	C40B—C39B—H39B	120.0
C12—C17—C16	120.0 (4)	C39B—C40B—C35B	120.0
C12—C17—H17	120.0	C39B—C40B—H40B	120.0
C16—C17—H17	120.0	C35B—C40B—H40B	120.0
			
N4—Co—N1—C1	159.7 (2)	C6—C7—C8—C34A	54.4 (7)
N2—Co—N1—C1	−16.4 (2)	C8—N4—C9—C10	176.6 (2)
Cl1—Co—N1—C1	−108.4 (2)	Co—N4—C9—C10	−44.8 (2)
Cl2—Co—N1—C1	72.1 (2)	C1—N1—C10—C9	177.0 (2)
N4—Co—N1—C10	−12.12 (17)	Co—N1—C10—C9	−10.5 (3)
N2—Co—N1—C10	171.72 (17)	N4—C9—C10—N1	35.8 (3)
Cl1—Co—N1—C10	79.72 (17)	N1—C1—C11—C12	−146.9 (3)
Cl2—Co—N1—C10	−99.78 (17)	C2—C1—C11—C12	36.6 (4)
N1—Co—N2—C4	−147.55 (17)	C1—C11—C12—C13	−100.9 (4)
N3—Co—N2—C4	33.62 (16)	C1—C11—C12—C17	76.4 (4)
Cl1—Co—N2—C4	−57.49 (16)	C17—C12—C13—C14	−0.8 (5)
Cl2—Co—N2—C4	122.50 (16)	C11—C12—C13—C14	176.6 (3)
N1—Co—N2—C3	−11.10 (19)	C12—C13—C14—C15	0.0 (5)
N3—Co—N2—C3	170.07 (19)	C13—C14—C15—C16	0.3 (6)
Cl1—Co—N2—C3	78.97 (18)	C14—C15—C16—C17	0.1 (7)
Cl2—Co—N2—C3	−101.05 (18)	C13—C12—C17—C16	1.2 (5)
N4—Co—N3—C6	−20.2 (2)	C11—C12—C17—C16	−176.2 (4)
N2—Co—N3—C6	156.0 (2)	C15—C16—C17—C12	−0.9 (7)
Cl1—Co—N3—C6	−112.1 (2)	N2—C3—C19—C20	−173.9 (2)
Cl2—Co—N3—C6	67.4 (2)	C18—C3—C19—C20	63.4 (3)
N4—Co—N3—C5	172.08 (17)	C2—C3—C19—C20	−59.5 (3)
N2—Co—N3—C5	−11.75 (17)	C3—C19—C20—C21	99.1 (3)
Cl1—Co—N3—C5	80.12 (16)	C3—C19—C20—C25	−83.0 (3)
Cl2—Co—N3—C5	−100.38 (16)	C25—C20—C21—C22	0.7 (4)
N1—Co—N4—C9	32.08 (16)	C19—C20—C21—C22	178.6 (3)
N3—Co—N4—C9	−149.10 (16)	C20—C21—C22—C23	−0.2 (5)
Cl1—Co—N4—C9	−57.59 (16)	C21—C22—C23—C24	0.0 (5)
Cl2—Co—N4—C9	122.43 (16)	C22—C23—C24—C25	−0.5 (5)
N1—Co—N4—C8	168.8 (2)	C23—C24—C25—C20	1.0 (5)
N3—Co—N4—C8	−12.35 (19)	C21—C20—C25—C24	−1.1 (4)
Cl1—Co—N4—C8	79.16 (18)	C19—C20—C25—C24	−179.0 (3)
Cl2—Co—N4—C8	−100.83 (18)	N3—C6—C26—C27	114.5 (3)
C10—N1—C1—C2	172.4 (2)	C7—C6—C26—C27	−60.0 (3)
Co—N1—C1—C2	1.0 (4)	C6—C26—C27—C28	117.1 (3)
C10—N1—C1—C11	−4.0 (4)	C6—C26—C27—C32	−62.3 (3)
Co—N1—C1—C11	−175.4 (2)	C32—C27—C28—C29	−0.6 (4)
N1—C1—C2—C3	47.3 (4)	C26—C27—C28—C29	179.9 (3)
C11—C1—C2—C3	−136.2 (3)	C27—C28—C29—C30	0.4 (5)
C4—N2—C3—C18	59.2 (3)	C28—C29—C30—C31	0.2 (5)
Co—N2—C3—C18	−73.0 (2)	C29—C30—C31—C32	−0.6 (4)
C4—N2—C3—C2	−179.6 (2)	C30—C31—C32—C27	0.4 (4)
Co—N2—C3—C2	48.2 (2)	C28—C27—C32—C31	0.2 (4)
C4—N2—C3—C19	−62.3 (3)	C26—C27—C32—C31	179.7 (2)
Co—N2—C3—C19	165.48 (17)	N4—C8—C34A—C35A	−175.9 (8)
C1—C2—C3—N2	−70.3 (3)	C33—C8—C34A—C35A	−54.6 (11)
C1—C2—C3—C18	51.6 (3)	C7—C8—C34A—C35A	66.0 (11)
C1—C2—C3—C19	174.0 (2)	C8—C34A—C35A—C36A	95.3 (10)
C3—N2—C4—C5	174.1 (2)	C8—C34A—C35A—C40A	−87.4 (9)
Co—N2—C4—C5	−48.0 (2)	C40A—C35A—C36A—C37A	0.0
C6—N3—C5—C4	178.9 (2)	C34A—C35A—C36A—C37A	177.3 (6)
Co—N3—C5—C4	−12.5 (3)	C35A—C36A—C37A—C38A	0.0
N2—C4—C5—N3	39.2 (3)	C36A—C37A—C38A—C39A	0.0
C5—N3—C6—C7	177.1 (2)	C37A—C38A—C39A—C40A	0.0
Co—N3—C6—C7	10.1 (4)	C38A—C39A—C40A—C35A	0.0
C5—N3—C6—C26	3.0 (4)	C36A—C35A—C40A—C39A	0.0
Co—N3—C6—C26	−163.99 (19)	C34A—C35A—C40A—C39A	−177.2 (6)
N3—C6—C7—C8	37.7 (4)	N4—C8—C34B—C35B	165 (2)
C26—C6—C7—C8	−147.7 (2)	C33—C8—C34B—C35B	−76 (3)
C9—N4—C8—C33	−62.9 (3)	C7—C8—C34B—C35B	43 (3)
Co—N4—C8—C33	164.00 (18)	C8—C34B—C35B—C36B	79 (3)
C9—N4—C8—C7	−178.2 (2)	C8—C34B—C35B—C40B	−95 (3)
Co—N4—C8—C7	48.7 (3)	C40B—C35B—C36B—C37B	0.0
C9—N4—C8—C34B	55.5 (17)	C34B—C35B—C36B—C37B	−173.2 (16)
Co—N4—C8—C34B	−77.6 (17)	C35B—C36B—C37B—C38B	0.0
C9—N4—C8—C34A	60.3 (7)	C36B—C37B—C38B—C39B	0.0
Co—N4—C8—C34A	−72.8 (6)	C37B—C38B—C39B—C40B	0.0
C6—C7—C8—N4	−66.1 (3)	C38B—C39B—C40B—C35B	0.0
C6—C7—C8—C33	177.7 (2)	C36B—C35B—C40B—C39B	0.0
C6—C7—C8—C34B	57.6 (19)	C34B—C35B—C40B—C39B	173.3 (15)

### Hydrogen-bond geometry (Å, °)

**Table:**

*D*—H···*A*	*D*—H	H···*A*	*D*···*A*	*D*—H···*A*
N2—H2n···O1	0.88 (3)	2.24 (3)	3.063 (4)	155 (2)
N4—H4n···Cl2^i^	0.88 (3)	2.64 (2)	3.432 (2)	150 (3)
C10—H10a···O3^ii^	0.99	2.50	3.437 (4)	159
C19—H19b···O1^iii^	0.99	2.54	3.409 (4)	147
C38a—H38a···O4^iv^	0.95	2.57	3.480 (3)	160

Symmetry codes: (i) −*x*, −*y*+1, −*z*+1; (ii) *x*−1, *y*, *z*; (iii) −*x*+1, −*y*, −*z*+1; (iv) *x*−1, *y*, *z*+1.
